# Subtemporal approach for posterior communicating artery aneurysms

**DOI:** 10.3389/fneur.2024.1518117

**Published:** 2024-12-19

**Authors:** Jing Lan, Xiao Huang, Yue Liu, Ting-bao Zhang, Jin-cao Chen, Zheng-wei Li

**Affiliations:** ^1^Department of Neurosurgery, Xiangyang No. 1 People’s Hospital, Hubei University of Medicine, Xiangyang, China; ^2^Department of Neurosurgery, Zhongnan Hospital of Wuhan University, Wuhan, China

**Keywords:** posterior communicating artery, aneurysm, subtemporal approach, clipping, pterional approach

## Abstract

**Background and objectives:**

Direct visualization of the aneurysmal neck and its related perforating arteries during microsurgical clipping of posterior communicating artery (PCoA) aneurysms with posterior projection or true PCoA aneurysms through the pterional approach may be difficult and complicated.

**Methods:**

From January 2022 to January 2023, the clinical and angiographic information regarding PCoA aneurysms were retrospectively collected. Among them, 10 consecutive patients with PCoA aneurysms treated with microsurgical clipping via the subtemporal approach in our single institution were included. Herein, we analyzed and summarized our experience and clinical outcomes to further evaluate the efficacy, safety and feasibility of this approach as well as the indications.

**Results:**

All aneurysms were completely clipped via the subtemporal approach. With respect to procedure-related complications, postoperative oculomotor nerve palsy occurred in one patient (10%), contralateral cerebral infarction in one patient (10%), and intraoperative rupture of the aneurysm in three patients (30%). There were no cases of temporal lobe contusion or venous injury in this group. Overall, Good outcomes were obtained in 9 patients (90%), and poor functional outcome was observed in 1 patient (10%) at the last follow-up.

**Conclusion:**

The management of true PCoA aneurysms and PCoA aneurysms projecting posteriorly is more complicated and challenging, and treating these lesions entails considerable risks via the pterional approach. Considering the above problems, we attempted to treat these refractory PCoA aneurysms through the subtemporal approach. Our results suggested that microsurgical clipping of these aneurysms via the subtemporal approach can achieve good clinical outcomes with a high preservation rate of the related branches. Appropriate patient selection, fully grasp of indications, precise understanding of the anatomy and thorough preoperative planning are crucial for successful surgery. The subtemporal approach appears to be a relatively safe and effective procedure in the experienced hands, and may be an alternative method for patients with true PCoA aneurysms or projecting posteriorly PCoA aneurysms which cannot be easily clipped from the pterional approach. It can provide a better lateral view to visualize the neck of the aneurysm, PCoA and its perforating vessels, as well as the other structures.

## Introduction

Posterior communicating artery (PCoA) aneurysms reportedly account for approximately 25% of intracranial aneurysms (1), usually originating from the junction of the internal carotid artery (ICA) and PCoA. However, an aneurysm that involves the PCoA itself, is located at some distance away from the junction of the ICA, which is known as a true PCoA aneurysm (2). A true PCoA aneurysm is relatively rare, with an incidence of approximately 0.1 to 3.6% (3, 4). PCoA aneurysms almost always arise from the superior aspect of the PCoA along the lateral surface of the ICA (5). PCoA aneurysms have various projection directions, and most of them can be clipped through the standard pterional approach. However, for true PCoA aneurysms and PCoA aneurysms projecting posteriorly, there are higher incidences of procedure-related complications than those with other origins and projections (6–8). Because the posterior surface of the ICA is challenging to directly visualize using the standard pterional approach, and the main body of aneurysms is obscured by the ICA, it is difficult to identify the PCoA and to confirm the patency of the parent artery and the related perforating arteries during clip placement. This makes microsurgical clipping more complicated via the standard pterional approach. Considering the above problems, we attempted to treat these refractory PCoA aneurysms by the subtemporal approach for a better view of the aneurysmal neck and other neurovascular structures. Here, we summarized our experiences and clinical outcomes of the subtemporal approach for microsurgical clipping these PCoA aneurysms, and to further evaluate the efficacy, safety and feasibility of this approach as well as the indications.

## Materials and methods

### Patient population

From January 2022 to January 2023, the clinical and angiographic information regarding PCoA aneurysms were retrospectively collected and analyzed. Patients with PCoA aneurysms projecting posteriorly or originating from the PCoA itself treated by microsurgical clipping via the subtemporal approach were included in this study. Patients with incomplete clinical data or treated by other surgical methods or approaches were excluded. A total of 10 patients with PCoA aneurysms met the above criteria in our institution were retrospectively reviewed. In general, treatment strategies and choices were discussed with a multidisciplinary team including neurosurgeons and neurointerventionists. The study was approved by the medical ethics committee of our hospital. Written informed consent for our procedures was obtained from individuals or their legal guardians.

Demographic data recorded for each patient included age, gender, initial clinical presentation, and functional status. All patients underwent three-dimensional computed tomography angiography (CTA) and digital subtraction angiography (DSA) to diagnose and evaluate the characteristics of PCoA aneurysms. The size of the aneurysm, aneurysmal neck, shape, rupture status of the aneurysm, presence of fetal-type PCoA, location and projecting direction, and surgical complications were detailed record. The size of the aneurysm was defined as the maximum measurement diameters of aneurysm height or aneurysm width. Projection directions and origin of the aneurysm were confirmed mainly according to the anteroposterior- and lateral-view carotid angiograms or three-dimensional DSA. Posterior-projecting aneurysms were previously defined as aneurysms projecting ≥45° posteriorly to the line perpendicular to the midline (6). The characteristics and clinical outcomes of PCoA aneurysms are described in Table 1.

**Table 1:** The characteristics and clinical outcomes of posterior communicating artery aneurysms.

Patient	Age (years), sex	Presentation	Status	Hunt–Hess grading system	GCS	Initial mRS	Size (mm)	Direction	Site of origin	Side	Intraoperative rupture	Complications	Postoperative mRS
1	66, F	Headache with nausea and vomit	Ruptured	1	15	1	2.37	Lateral	PCoA itself	Left	Yes	Infarction	3
2	63, F	Headache and vomit	Ruptured	1	15	1	3.71	Posterior	ICA-PCoA junction	Left	No	No	1
3	77, M	Headache with ptosis	Unruptured	0	15	1	5.85	Posterior	ICA-PCoA junction	Left	Yes	No	1
4	65, F	Dizziness	Unruptured	0	15	1	3.75	Posterior	ICA-PCoA junction	Left	No	Oculomotor nerve palsy	1
5	73, F	Headache	Ruptured	3	14	1	3.11	Posterior	ICA-PCoA junction	Left	No	No	1
6	68, F	Dizziness	Unruptured	0	15	1	3.57	Posterior	ICA-PCoA junction	Right	No	No	1
7	71, F	Headache	Ruptured	1	15	1	5.32	Posterior	ICA-PCoA junction	Left	Yes	No	1
8	67, F	Dizziness	Unruptured	0	15	1	1.83	Posterior	ICA-PCoA junction	Right	No	No	1
9	72, F	Headache	Ruptured	3	14	1	3.91	Posterior	ICA-PCoA junction	Left	No	No	1
10	65, M	Nausea	Ruptured	1	15	1	4.31	Posterior	ICA-PCoA junction	Right	No	No	1

GCS, Glasgow Coma Scale; ICA, internal carotid artery; mRS, Modified Rankin Scale; PCoA, posterior communicating artery.

### Subtemporal approach

The patient was placed supine on the table with a shoulder pillow beneath the ipsilateral shoulder. The head was rotated until the temporal region lay horizontal with the vertex down, then slightly drooped and overall elevated above the heart level. A modified straight incision was made from the base of the zygomatic arch to the parietal tuber. The skin and temporalis muscle were stratified dissection, the temporalis muscle was pulled sideways to expose the skull. A subtemporal craniotomy was performed. After the removal of the bone flap, part of the bone from the temporal squama to the floor of the temporal fossa was fully removed to expose the base of the middle cranial fossa. The dura was then opened to expose the surface of the temporal lobe. Under an operating microscope, the temporal lobe was retracted with malleable brain retractors to expose the free margin of the tentorium. The arachnoid covering the perimesencephalic cistern was meticulously incised to further release cerebrospinal fluid and allow brain relaxation. The course of ICA, PCoA, and its relation to the position of aneurysm were identified. Dissect and expose the proximal ICA for the proximal control to prevent intraoperative uncontrollable aneurysmal hemorrhage. A part of the aneurysm could be covered by the tentorial edge, in order to obtain sufficient surgical exposure and wider operating space, we need to incise the tentorium. The aneurysmal neck is clipped by a straight or bayonet aneurysm clip. In the process of aneurysm clipping, the PCoA and its related perforators should be avoided from being injured or accidentally clipped. After the clipping of the aneurysm, the dura membrane was tightly sutured. The bone flap was restored and affixed, and the temporalis muscle and scalp were sutured by layers.

### Outcome assessment

To evaluate the efficacy of intraoperative observation of the aneurysm and preservation of the relevant branches, complete occlusion of the aneurysm, and procedural-related complications by the subtemporal approach. The results of surgical treatment were assessed based on intraoperative indocyanine green angiography (ICGA) and postoperative cerebral angiography, and then graded as completely or partially clipped. Postoperative complications were recorded, a hemorrhagic complication was confirmed by postoperative computed tomography (CT), and magnetic resonance imaging (MRI) was performed to evaluate ischemic lesions. Follow-up DSA or CTA was performed to evaluate the recurrence of the aneurysm. Functional status was assessed by the modified Rankin Scale (mRS), the pre-operative mRS and follow-up information was obtained from hospital admission physical examination and hospital records. We noted the mRS score at the time of each visit and angiography. A good outcome was defined as a mRS score of 0–2 at the last clinical follow-up and a bad outcome as a mRS score of 3–6.

### Statistical analysis

All statistical analysis was performed with the standard software (SPSS v23; SPSS, Chicago, IL). Continuous data was expressed as mean ± standard deviation (SD). The frequencies and percentages of categorical variables are reported.

## Results

These 10 patients comprised 2 men (20%) and 8 women (80%), with a mean age of 68.70 ± 4.40 years (ranging from 63 to 77 years). The majority of patients (*n* = 6; 60%) presented with subarachnoid hemorrhage (SAH), of which 4 were Hunt–Hess grade I, 2 were grade III, all of them had a good neurologic grade (World Federation of Neurosurgical Societies grade I to II). Of the remaining 4 (40%) patients with unruptured aneurysms, 1 presented with headache with oculomotor nerve palsy, and 3 presented with dizziness. There was no aneurysmal SAH with associated intracranial hematoma cases in this group. There were 7 aneurysms located on the left side, and 3 on the right. The aneurysms ranged in size from 1.83 to 5.85 mm, with an average diameter of 3.77 ± 1.21 mm. There were 8 small (<5 mm) and 2 medium (5–10 mm) aneurysms. One aneurysm arises from the proximal portion of the PCoA itself, and the remaining nine aneurysms originate from the junction of the ICA and PCoA. Among these patients, 9 PCoA aneurysms project posteriorly and 1 true PCoA aneurysm laterally. All aneurysms were completely clipped via the subtemporal approach. The course of PCoA and its related perforating arteries were intraoperatively identified and preserved in all cases. With respect to the procedure-related complications, postoperative oculomotor nerve palsy occurred in one patient (10%), contralateral cerebral infarction in one patient (10%), and intraoperative rupture of the aneurysm in three patients (30%). Among the 3 aneurysms that ruptured during the operation, 1 was a small-size ruptured true PCoA aneurysm and 2 were medium-size PCoA aneurysms. Of the two medium-sized aneurysms, one was ruptured aneurysm and the other was unruptured aneurysm. Unfortunately, the patient with cerebral infarction was discharged from the hospital with neurological deficits. The initial mRS score of the patient was 1, and the postoperative neurological status deteriorated, with an mRS score of 3. The patient with oculomotor nerve palsy completely recovered within 1 month after surgery. There were no cases of temporal lobe contusion and venous injury after surgery in this group. Overall, Good outcomes were obtained in 9 patients (90%), and poor functional outcome was observed in 1 patient (10%) at the last follow-up. All aneurysms were completely occluded, and no recurrence of PCoA aneurysms or new neurological deficits occurred during the follow-up period.

### Illustrative cases

**Figure 1:**
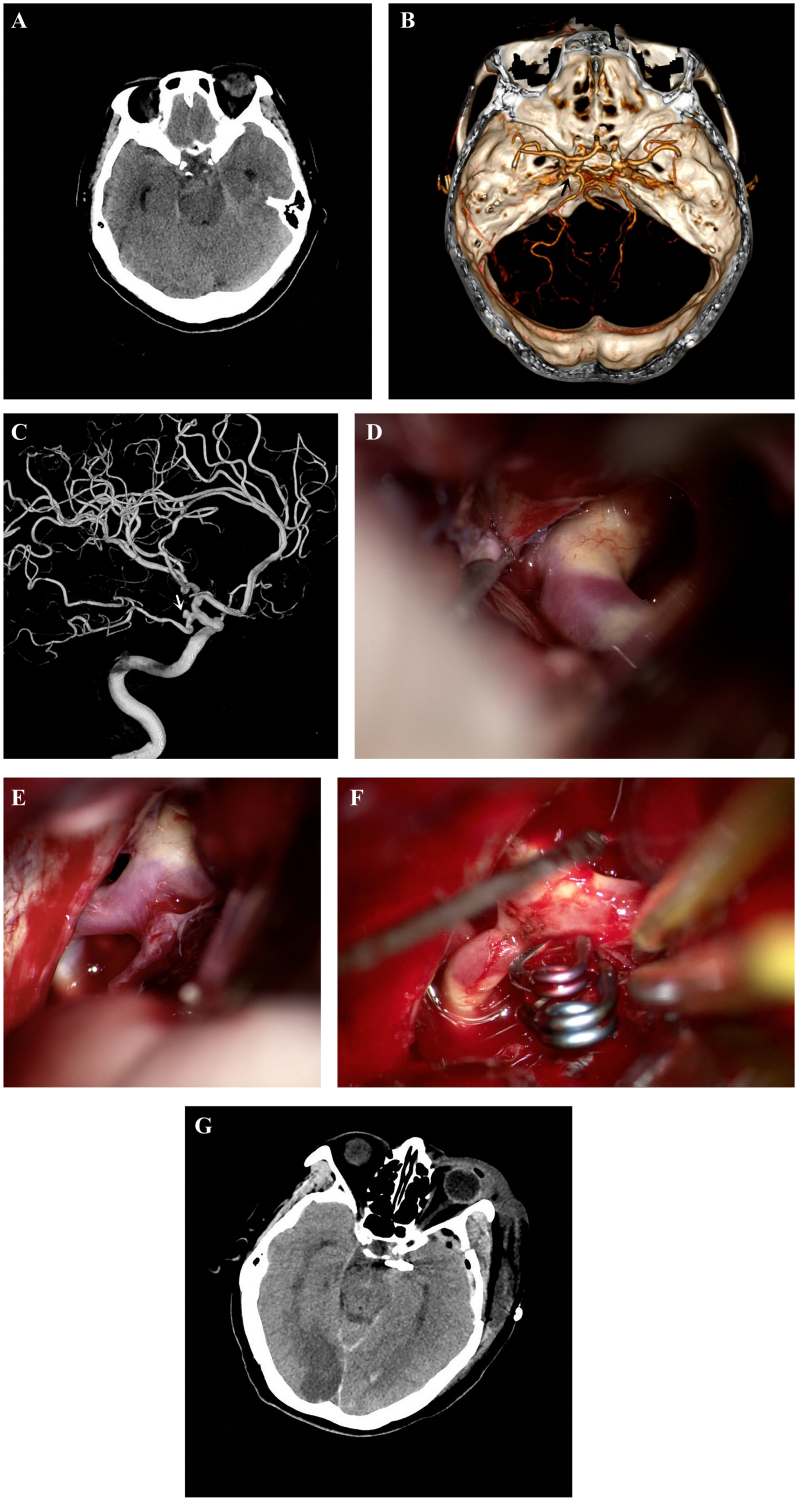
Patient 1. Head CT **(A)** showed a diffuse SAH in the basilar cistern. CTA **(B)** revealed the laterally projected PCoA aneurysm (black arrow) on the left side. Preoperative three-dimensional DSA **(C)** of the left ICA manifested that the aneurysm (white arrow) was about 2.37 × 1.68 mm, arising from the PCoA itself, a few millimeters away from the junction with ICA. Intraoperative observation **(D)** from the standard pterional approach showed that the greater part of the aneurysm was hidden behind the ICA and the left temporal lobe. The subtemporal approach **(E)** offers a more lateral line of vision to well visualize the aneurysmal neck and the PCoA. The aneurysm was successfully and safely obliterated with two straight titanium clips (Yasargil titanium clip; No. FT710T and No. FT740T; Aesculap AG, Tuttlingen, Germany) **(F)**. The postoperative head CT **(G)** disclosed right occipital lobe infarction and no secondary hemorrhage. CT, computed tomography; CTA, computed tomography angiography; DSA, digital subtraction angiography; ICA, internal carotid artery; PCoA, posterior communicating artery; PCA, posterior cerebral artery; SAH, subarachnoid hemorrhage.

Patient 1 (Figure 1) was a 66-year-old woman who suddenly suffered a headache with nausea and vomit for one day. Her past medical history was unremarkable, and she did not take any regular medication. Neurological examinations at admission showed that the Hunt–Hess grade was I. Head CT (Figure 1A) showed a diffuse SAH in the basilar cistern, and CTA (Figure 1B) revealed the laterally projected PCoA aneurysm on the left side. Preoperative three-dimensional DSA of the left ICA (Figure 1C) manifested that the aneurysm was about 2.37 × 1.68 mm, arising from the PCoA itself, a few millimeters away from the junction with ICA. The subtemporal approach combined with the pterional approach was performed for this ruptured true PCoA aneurysm. We observe the morphology, position and projection of the aneurysm under different surgical approaches. After the Sylvian cistern was dissected and opened, the ICA was exposed. We observed that the aneurysm was approached through the standard pterional approach but could not be visualized well, and the PCoA and its perforating arteries were not clearly shown, because the greater part of the aneurysm was hidden behind the ICA and the left temporal lobe (Figure 1D). Surgical clipping of the aneurysm through the standard pterional approach may result in a residual aneurysm neck in the shape of “dog ear” and incomplete obliteration of the aneurysm. Anterior clinoid process may need to be drilled to increase operative space for clipping. A more lateral approach than the pterional approach may be thus required to clip this aneurysm. Then we retracted the temporal lobe and cut the arachnoid trabeculae via the subtemporal approach to expose the aneurysm and confirm the origin and course of the PCoA. The subtemporal approach offers a more lateral line of vision and allows the surgeon to well visualize the aneurysmal neck and the PCoA (Figure 1E). During the operation, we found the dome of the aneurysm projected laterally. The aneurysm was ruptured when dissecting and separating the neck of the aneurysm. Under direct visual control and point-suction, the bleeding was effectively controlled, and the aneurysm was successfully obliterated with two straight titanium clips (Yasargil titanium clip; No. FT710T and No. FT740T; Aesculap AG, Tuttlingen, Germany) (Figure 1F). Intraoperative ICGA demonstrated complete occlusion of the aneurysm and the patency of both the PCoA and its perforating arteries. The postoperative head CT (Figure 1G) revealed right occipital lobe infarction and no secondary hemorrhage. The initial mRS score of the patient was 1, and the postoperative neurological status deteriorated, with an mRS score of 3. We speculate that the cause of cerebral infarction may be associated with severe stenosis of the P2 segment of the right PCA and vasospasm. Unfortunately, the patient was discharged from the hospital with neurological deficits.

**Figure 2:**
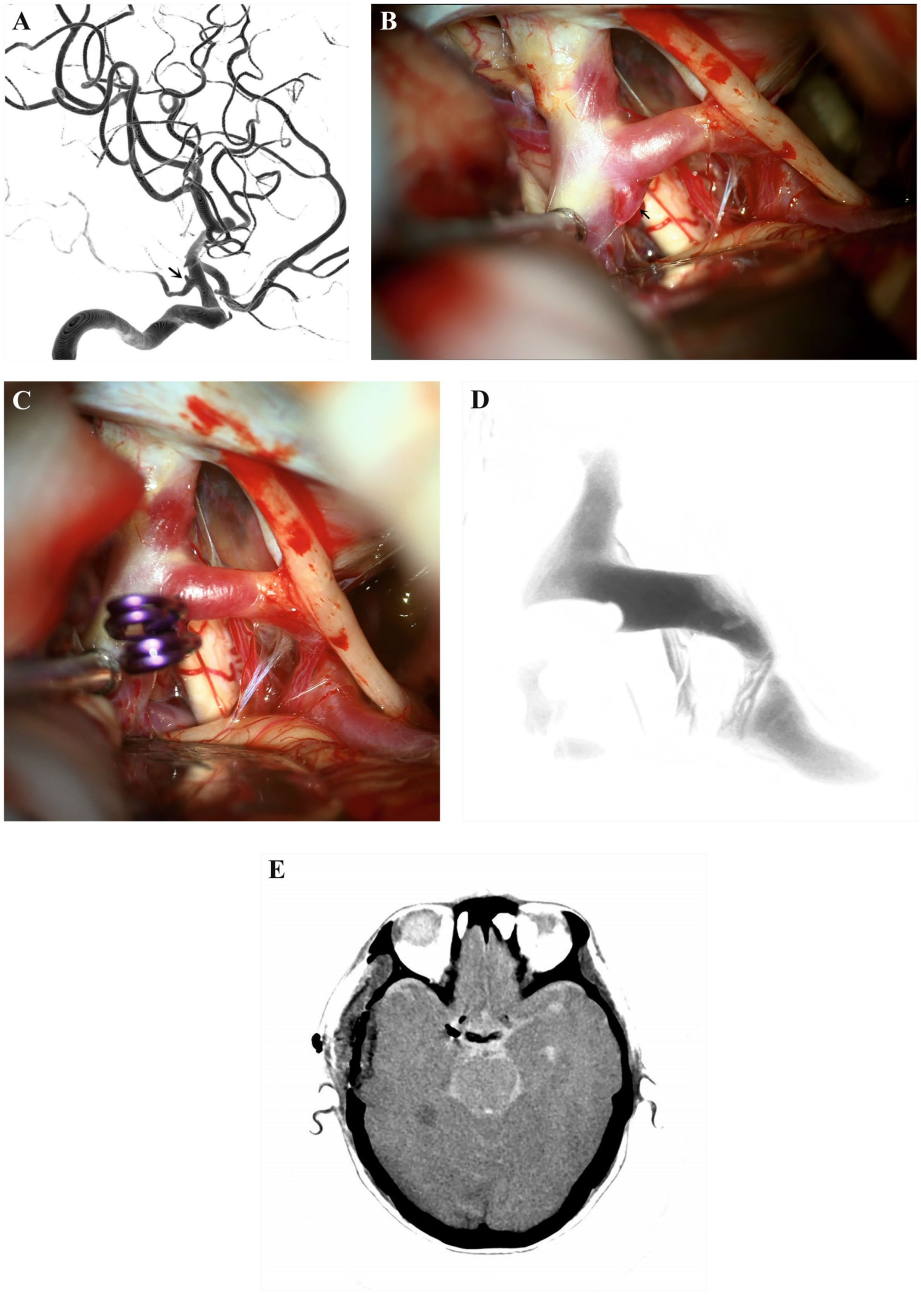
Patient 8. Preoperative DSA **(A)** with lateral view of the right ICA showed a saccular aneurysm (white arrow) arising from the communicating segment of the left ICA. The aneurysm is about 1.02 × 31.83 mm in size, and the dome of the aneurysm is projected posteriorly. The aneurysm (black arrow) was approached and well visualized through the subtemporal approach **(B)**. The aneurysm was completely clipped with two straight titanium clips (Yasargil titanium clip; No. FT710T; Aesculap AG, Tuttlingen, Germany) **(C)**. Intraoperative ICGA **(D)** confirmed that the aneurysm is clipping completely and patency of the PCoA and perforating arteries. The postoperative head CT **(E)** showed no cerebral infarction, temporal lobe contusion and secondary hemorrhage. CT, computed tomography; DSA, digital subtraction angiography; ICA, internal carotid artery; ICGA: indocyanine green angiography; PCoA, posterior communicating artery.

Patient 8 (Figure 2) was a 67-year-old woman who suffered dizziness over 2 years. She had a 10-years history of hypertension and hyperlipidemia. On admission, neurological examinations revealed no focal neurological deficits. Initial head CT showed no evidence of intracranial hemorrhage and infarction. Preoperative three-dimensional DSA with a lateral (Figure 2A) view of the right ICA showed an aneurysm arising from the communicating segment of right ICA. The saccular aneurysm is about 1.02 × 1.83 mm in size, and the dome of the aneurysm is projected posteriorly. The patient underwent the right subtemporal approach for microsurgical clipping. The temporal lobe was retracted and the arachnoid was dissected to expose the aneurysm. The aneurysm was approached and well visualized through the subtemporal approach (Figure 2B). Two straight titanium clips (Yasargil titanium clip; No. FT740T; Aesculap AG, Tuttlingen, Germany) were used to ensure complete occlusion of the aneurysm (Figure 2C). Finally, intraoperative ICGA (Figure 2D) was applied to confirm the aneurysm clipping completely and patency of the PCoA and perforating arteries. The postoperative course of the patient was uneventful, and head CT (Figure 2E) showed no cerebral infarction, temporal lobe contusion and secondary hemorrhage. The patient was discharged without any neurological deficits. The aneurysm was successfully obliterated, and there were no *de novo* aneurysms during follow-up angiograms.

## Discussion

Intracranial aneurysms tend to occur at the bifurcation of the cerebral arteries, and the PCoA junction is the most common site (5). The goal of treatment for these PCoA aneurysms is to obliterate the aneurysm as well as preserve the PCoA and the related branches. The success rate of microsurgical clipping is associated with precise anatomical knowledge, well-organized surgical planning and appropriate surgical approach. For PCoA aneurysms, multiple surgical approaches can be adopted. Although each of the PCoA aneurysms is unique and may require modification of the surgical techniques and approaches based on different anatomical variations, the general principles for safe microsurgical clipping are the same. Therefore, it is of great importance to evaluate the aneurysmal size, location, direction, shape, and neck of the aneurysm as well as the relationship between the aneurysm and parent artery and perforator vessels in detail before formulating the optimal treatment strategies and surgical approach. The size, position and direction of PCoA aneurysm projection may directly affect the choices of surgical approaches.

In this study, we presented a series of PCoA aneurysms projecting posteriorly or originating from the PCoA itself. For these cases, it needs to visualize the aneurysmal neck through the retrocarotid space. In the pterional approach, it allows for a wide range of inclinations for the clip-applying forceps. When microsurgery is performed to clip these PCoA aneurysms via the pterional approach, the PCoA and its associated branches may be compromised, which requires more complex and sophisticated clipping techniques with fenestrated clips or a more lateral surgical exposure. However, the postoperative complications of PCoA aneurysms projecting posteriorly or originating from the PCoA itself are significantly higher than those PCoA aneurysms with lateral projection or at other sites (7, 9). Because PCoA aneurysms with posterior projection are more likely to adhere to the PCoA or its associated perforating arteries, the application of fenestrated clips may unintended clip the perforating arteries, and true PCoA aneurysms often involving the fetal PCoA, which are more prone to cause intraoperative rupture during the dissection of these aneurysms (7, 9). Moreover, true PCoA aneurysms may be located at the infundibular dilatation of the PCoA or close to the ICA, at the middle part of the PCoA, or proximal to the PCA, and the whole aneurysm or most of the aneurysm may be buried in the temporal lobe. Thus, it is useful and necessary to retract the temporal lobe via the pterional approach during the exposure of the aneurysmal neck, which may lead to aneurysm premature rupture and bleeding. However, intraoperative bleeding cannot be sufficiently controlled by the temporary clip of the proximal ICA or fetal PCoA alone for true PCoA aneurysms, as the blood flow can be refluxed from posterior circulation via distal fetal PCoA. Intraoperative rupture of PCoA aneurysms and uncontrolled bleeding may cause serious complications. Therefore, we try to adopt the subtemporal approach rather than the pterional approach to obliterate these refractory aneurysms.

The subtemporal approach was first proposed by Drake for the treatment of basilar artery aneurysms (10). The subtemporal approach is more laterally positioned relative to the pterional approach, We performed the preoperative CTA or fusion image of three-dimensional DSA and Xper-CT to evaluate the position relationship between aneurysms, parent artery, perforating arteries and anterior clinoid process. Aneurysms were placed at the pterional approach and the subtemporal approach respectively, and then we found that it was difficult to completely expose and visualize the neck of the aneurysm as well as the PCoA and its perforating arteries in the pterional approach. In order to achieve a satisfactory surgical visualization, the angle of the operating microscope can be adjusted or the patient’s head can be rotated to the contralateral side. In addition, an endoscope or even a surgical mirror can be used to observe the aneurysm before clip application and to detect any remaining neck of the aneurysm after clip application. But in fact, these devices cannot improve the available space in a narrow retrocarotid space (11). Thus, to obtain a wider operating space for the exposure and occlusion of the aneurysm through the standard pterional approach, we may need to drill off the anterior clinoid process and open the optic nerve canal, then incise carotid dura ring to mobilize the ICA and optic nerve. The subtemporal approach provided the more better lateral view, it may be possible to visualize the whole length of the PCoA, and its perforating vessels as well as a better observation of the neck of the aneurysm, which is facilitated to block the parent vessels, clip the aneurysms, and reduce postoperative complications. It also provided a short operative distance and a direct trajectory to the aneurysm, with less dissection to expose the aneurysm, which helps to protect the perforating arteries. When the temporal lobe was progressively retracted and the arachnoid was sharply dissected, the ipsilateral tentorium cerebelli may interfere with the visibility and manipulation around the aneurysmal neck. So in some cases, we need to cut and divide the tentorium cerebelli to further obtain a sufficient operative field and expose the neck of the aneurysm. For the present series of cases, the tentorium cerebelli was cut in four of the patients. It’s worth noting that the aneurysms are located around or under the tentorial edge. What’s more, the excessive retraction of the temporal lobe will increase the potential risk of temporal lobe contusion and vein injury. The rate of temporal lobe contusion is approximately 14% in other surgical approaches for PCoA aneurysms (12). Fortunately, there were no cases of temporal lobe contusion and venous injury in the current study. During the procedure of aneurysm exposure, adequate release and drainage of cerebrospinal fluid to achieve sufficient brain relaxation, can effectively reduce these risks. In this group of cases, we did not perform external ventricular drainage or lumbar cisterna drainage in advance, and we only released cerebrospinal fluid intraoperatively in a stepwise manner to reduce intracranial pressure. In the present series, one patient presented with preoperative oculomotor nerve palsy, which may be related to the compression of the oculomotor nerve caused by the tiny enlargement or morphological change of the PCoA aneurysm, and another patient (10%) developed transient left oculomotor nerve palsy after surgery, which may be attributable to the separation and traction of the oculomotor nerve or the bipolar coagulator related heat injury. Because the oculomotor nerve has a close relationship to the aneurysm and usually runs below and lateral to the PCoA, and some PCoA aneurysms protruding posteriorly may adhere to the oculomotor nerve, which is prone to cause intraoperative injury. We should be aware of the increased risk of oculomotor nerve injury through the subtemporal approach in comparison with the pterional approach, and should accurately identify the oculomotor nerve and perform sharp dissection of the arachnoid around the nerve as much as possible. The rate of oculomotor nerve palsy after surgery in other series of subtemporal approaches is approximately 34% (13), and the palsy usually subsides over weeks to months. During the follow-up, the patient’s ptosis symptoms were completely relieved. Several important perforating branches arise from the PCoA and supply the optic tract, oculomotor nerve, thalamus, hypothalamus, caudate nucleus, internal capsule and other critical structures (3, 9). The PCoA aneurysm with protruding posteriorly was more likely to adhere to these important branches, and the anterior choroidal artery (AChA) runs very close or attaches to the distal neck of the PCoA aneurysm. The inadvertent injury or occlusion of PCoA, its related perforating arteries and AChA during microsurgical clipping may cause severe neurological deficits, such as hemiparesis and consciousness disturbance. Therefore, it is necessary to preserve and confirm the patency of these vessels, especially when the PCA and PCoA are of fetal origin. These vessels are clearly visible under direct visualization and easily protected via the subtemporal approach. In addition to the extent of surgical exposure of an aneurysmal neck and the preservation of perforating vessels, the controlling of the parent artery is also the key to the successful clipping of aneurysm. Intraoperative uncontrollable premature rupture of PCoA aneurysms is the most catastrophic event, which may lead to massive hemorrhage or cause cerebral ischemia by prolonged application of temporary clip or damage the surrounding tissue in the limited operative field. Temporary clipping of the parent artery can effectively reduce intra-aneurysm pressure, which facilitates the dissection of the aneurysm and reduces the risk of rupture and uncontrollable bleeding (14). The subtemporal approach has a relatively short operative distance and a wide visual field, which is conducive to the temporary clipping of the parent artery. Intraoperative rupture of the aneurysms occurred in three patients and temporary clipping was used in four cases in our cohort, the primary reason for avoiding using temporary clipping was the calcification of the proximal ICA. Intraoperative exposure of the carotid artery from the neck may be necessary for proximal arterial control in these patients with significant ICA calcification. Among the 3 aneurysms that ruptured during the operation, 1 was a small-size ruptured true PCoA aneurysm and 2 were medium-size PCoA aneurysms. Of the two medium-sized aneurysms, one was ruptured aneurysm and the other was unruptured aneurysm. We may assume that the size of the aneurysm is related to intraoperative aneurysm rupture and procedure-related complications, since large aneurysms require more extensive surgical exposure and a wider surgical corridor, increasing the risk of aneurysm rupture. After the aneurysms were successfully clipped, the patency of the PCoA and the related perforator vessels was confirmed mainly by ICGA. In our report, all patients achieved complete occlusion of aneurysm, but only one patient (10%) presented with postoperative contralateral occipital lobe infarction without secondary hemorrhage, which may be associated with severe stenosis of the P2 segment of the right PCA and vasospasm. We considered the subtemporal approach is more suitable for small-size PCoA aneurysms projecting posteriorly and true PCoA aneurysms with different projections than the standard pterional approach. Although this approach provides a more lateral vision for good visualization of the course of the PCoA and its perforators, it carries the potential risk of temporal lobe contusion and venous injury, as well as oculomotor nerve injury.

### Limitations

The number of patients included in this research is relatively small and this study was conducted in a retrospective manner, which may cause some bias. There was no available comparative group to further evaluate the benefits of the subtemporal approach for these aneurysms. In addition to the characteristics of aneurysms, many other factors may affect the choice of surgical approaches, which were not evaluated and discussed in detail in this paper. As stated above, further studies and larger case series may be necessary to assess the efficacy and confirm the durability of this treatment.

## Conclusion

The management of true PCoA aneurysms and PCoA aneurysms projecting posteriorly is more complicated and challenging, and treating these lesions entails considerable risks via the pterional approach. Considering the above problems, we attempted to treat these refractory PCoA aneurysms through the subtemporal approach. Our results suggested that microsurgical clipping these aneurysms via the subtemporal approach can achieve good clinical outcomes with a high preservation rate of the related branches. Appropriate patient selection, fully grasp of indications, precise understanding of the anatomy and the hazards associated with PCoA aneurysms, as well as thorough preoperative planning are crucial for successful surgery. The subtemporal approach appears to be a relatively safe and effective procedure in the experienced hands, and may be an alternative method for patients with true PCoA aneurysms or projecting posteriorly PCoA aneurysms which cannot be easily clipped from the pterional approach. It can provide a better lateral view to visualize the neck of the aneurysm, PCoA and its perforating vessels, as well as the other structures.

## Data Availability

The original contributions presented in the study are included in the article/supplementary material, further inquiries can be directed to the corresponding authors.
